# Distribution and Assembly Processes of Soil Fungal Communities along an Altitudinal Gradient in Tibetan Plateau

**DOI:** 10.3390/jof7121082

**Published:** 2021-12-16

**Authors:** Sarfraz Hussain, Hao Liu, Senlin Liu, Yifan Yin, Zhongyuan Yuan, Yuguo Zhao, Hui Cao

**Affiliations:** 1 Key Laboratory of Agricultural Environmental Microbiology, Ministry of Agriculture and Rural Affairs, College of Life Sciences, Nanjing Agricultural University, Nanjing 210095, China; 2017216037@njau.edu.cn (S.H.); 2020816105@stu.njau.edu.cn (H.L.); 2019216014@njau.edu.cn (S.L.); 2018216014@njau.edu.cn (Y.Y.); 2018116041@njau.edu.cn (Z.Y.); 2 State Key Laboratory of Soil and Sustainable Agriculture, Institute of Soil Science, Chinese Academy of Sciences, Nanjing 210008, China; ygzhao@issas.ac.cn

**Keywords:** fungal community, Tibetan Plateau, stochastic processes, dispersal limitations, βNTI, neutral processes, niche processes

## Abstract

In soil ecosystems, fungi exhibit diverse biodiversity and play an essential role in soil biogeochemical cycling. Fungal diversity and assembly processes across soil strata along altitudinal gradients are still unclear. In this study, we investigated the structure and abundance of soil fungal communities among soil strata and elevational gradients on the Tibetan Plateau using Illumina MiSeq sequencing of internal transcribed spacer1 (ITS1). The contribution of neutral and niche ecological processes were quantified using a neutral community model and a null model-based methodology. Our results showed that fungal gene abundance increased along altitudinal gradients, while decreasing across soil strata. Along with altitudinal gradients, fungal α-diversity (richness) decreased from surface to deeper soil layers, while β-diversity showed weak correlations with elevations. The neutral community model showed an excellent fit for neutral processes and the lowest migration rate (R^2^ = 0.75). The null model showed that stochastic processes dominate in all samples (95.55%), dispersal limitations were dominated at the surface layer and decreased significantly with soil strata, while undominated processes (ecological drift) show a contrary trend. The log-normal model and the null model (βNTI) correlation analysis also neglect the role of niche-based processes. We conclude that stochastic dispersal limitations, together with ecological drifts, drive fungal communities.

## 1. Introduction

Biodiversity plays a central role in ecosystem functioning and understanding changes in biodiversity is a central focus in ecology. Species in montane regions are frequently cited as being particularly vulnerable to the effects of climate change. Mountain ecosystems have unique edaphic, climatic conditions, and vegetation types even over small spatial extents. Thus, these altitudinal gradients provide “natural experiments” to study biological diversity patterns [1]. Over the past century, variations in biodiversity along altitudinal gradients have been studied with great interest [2,3]. About 250 years ago, Linnaeus and his contemporaries observed diversity patterns of plants and animals along altitudinal gradients, and these observations had a significant impact on the development of ecology and biogeography [4]. Previous studies of generalized altitudinal patterns of biodiversity answered the question of how individual species and/or community composition responded to altitudinal gradients [5,6]. The assembly processes and mechanisms that shape the community distributions of plants and animals along with altitudinal gradients have been investigated [7,8]. However, limited studies have focused on microbial diversity along altitudinal gradients and little is known about how the microbial community is assembled across elevation gradients [9,10]

A common framework to understand assembly processes is the distinction between deterministic (niche) and stochastic (neutral) processes that shape communities [11,12]. Initially, it was assumed that due to short generation time, large population size, faster growth, and high dispersal rate of microorganisms traditionally assembled by deterministic processes. However, in recent decades, microbial communities have provided evidence that in addition to environmental heterogeneity, stochastic processes can be important drivers of community assembly processes [13,14,15]. Community assembly processes can be categorized easily, such as demographic drift, death and birth rate, as well as population size as purely stochastic processes [16]. Species sorting and environmental filtering are totally deterministic processes, while on the other hand, dispersal and diversity can be either deterministic or stochastic [17,18]. The degree of dispersion within a meta-community can range from a very low to a very high degree of community turnover. Low levels of dispersal inhibit the turnover of microbes between local communities, leading to the geographic fluctuation in composition; this is referred to as “dispersal limitation.” Limiting dispersal, on the other hand, is insufficient to generate geographic turnover in the community’s composition. The structure of ecological communities may diverge as a result of stochastic variations in population size due to the limited exchange of organisms between small communities. As a result, dispersal limitation facilitates drift to create a considerably greater geographic variation in community composition than drift alone [19].

Fungi are important soil eukaryotic microorganisms that drive a variety of vital ecosystem functions such as litter decomposition, nutrient cycling, and plant growth control [20]. In recent years, the development of modern molecular methods has led to the growth in the number of studies on fungal diversity patterns along elevation gradients [21,22]. Most of these studies indicate that fungal diversity can be influenced by the environment along with altitudinal gradients. For example, Want et al. reported that fungal diversity along elevation was governed by pH [23]. The ectomycorrhizal fungi have shown a humped-shaped diversity pattern along altitudinal gradients of mount Fuji ranging from 1100–2250 m [3]. In addition, non-significant distribution patterns of fungal communities were also reported [24]. Regarding subsurface diversity and assembly processes of soil fungi and bacteria, it has been suggested that diversity from surface to subsurface may differ [25].

The Tibetan Plateau is the largest and highest plateau in the world (average altitude 4500 m above sea level). It covers an area of 2.61 × 10^6^ km^2^ in western China and includes Tibet and Qinghai provinces, as well as some areas of Xinjiang, Gansu, Sichuan, and Yunnan provinces [26]. Furthermore, the Tibet Plateau is distinguished by unique weather and climate factors due to its complex terrain and diverse boundary conditions [27]. Summers are hot and humid, while winters are cool and dry, with an average annual temperature of 1.61 °C and 413.6 mm of precipitation [28]. Therefore, the Tibetan Plateau serves as an excellent natural laboratory for researchers interested in regional microbial distribution patterns. Due to the mostly non-anthropogenic conditions, this area is suitable for studying natural soil microbial distributions and the ecological processes that influence community structure and composition at various soil layers [29]. The current study focused on the Tibetan Plateau because human activity is too rare, and there are very typical plateau landscapes along the mountain [23]. Several studies (listed below) on the Tibetan Plateau were conducted to investigate microbial community composition and structure, including an investigation on the contemporary environment affecting the bacterial community composition between the surface (0–15 cm) and subsurface (15–30 cm) soil layers [29]. Another recent study showed that bacterial community composition varied according to elevation and land use [30]. Other studies on the Tibetan Plateau mainly focused on freshwater lakes and saline lakes to investigate microbial community composition and diversity patterns [31,32,33,34]. To our knowledge, this is the first comprehensive study to investigate fungal community composition, diversity, abundance, and assembly mechanisms along altitudinal gradients in three different soil layers on Tibetan Plateau. In addition, we compared the composition of fungal communities, taxonomic, phylogenetic diversity patterns, and assembly mechanisms with quantified environmental variables and variations in nutrient availability in different soil layers along altitudinal gradients.

This study addressed four questions: (1) what is the composition and structure of fungal communities along altitudinal gradients? (2) How do fungal diversity, richness, and abundance vary along elevation and different soil layers? (3) What are the assembly processes that govern fungal community composition along elevation? (4) What are the major factors (spatial and/or environmental) that influence the community structure and assembly processes of fungi? To answer these questions, we collected soil samples from seven different altitudes (889 m–3787 m), soil samples were collected from each altitude surface and subsurface (0–100 cm). The fungal abundance was investigated by using a quantitative polymerase chain reaction (qPCR). The community structure and composition of fungi were analyzed by using the Illumina sequencing platform. The neutral community model of Sloan et al. [35] was used to assess the impact of neutral or stochastic processes in driving the biogeography of the fungal community. To determine the influence of environmental and/or spatial distance on fungal diversity and assembly processes, we performed the Mantel test.

## 2. Materials and Methods

### 2.1. Overview of the Study Area and Soil Sample Collection

**Figure 1:**
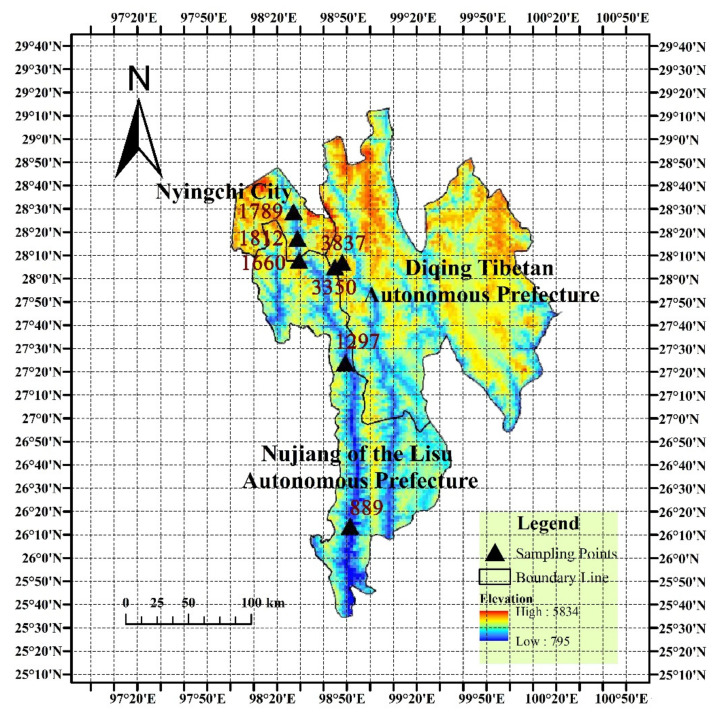
Location map of sampling sites in the Tibetan Plateau.

This study was conducted on the Tibet Plateau (98°27′41″ E–98°52′4″ E, 26°12′39″ N–28°6′33″ N), which is the largest and highest plateau in the world with an average elevation of over 4000 m. The soil types in this study area were brown, Calcareous cinnamon, and Frozen embryonic soil. Land cover types were defined based on field evidence, including vegetation cover and dominant species (trees, shrubs, herbs). The elevation of sampling sites ranged from 889 m to 3837 m and the latitude from 26.12 to 50° W. Along the altitudinal gradient, there were mountainous temperate zones from 889 to 3350 m sampling site, subalpine cool temperate zones between altitudes 3350 and 3837 m sampling site. The sampling site description is shown in Table S1, Figure 1. A standard protocol was used throughout sampling and transport to avoid any contamination [36,37]. The soil layers in this study were classified according to changes in soil appearance (i.e., color and structure): surface (0–20 cm), middle (20–60 cm), deep (60–100 cm). From each altitude, 9 soil subsamples were collected belonging to 3 different depths, where each depth was represented by 3 replicates. Soil samples were collected at a depth interval of 0–20 cm, 20–60 cm, and 60–100 cm, and the distance between replicates did not exceed 100 m. The entire soil profiles were collected with a spade. For each replicate, at least 300 g of soil was collected from the bottom to upper layers.

A total of 63 soil samples were collected from 7 different altitudes, each represented by 3 different depths, and then kept in sterilized plastic zipper bags at the study location. Soil samples were transferred on ice packs to Key Laboratory of Agricultural Environmental Microbiology, Ministry of Agriculture and Rural Affairs, College of Life Sciences, Nanjing Agricultural University, Nanjing, China, for physicochemical analysis and imminent microbial analysis. Roots, stones, and other residues were removed from soil samples by grinding them and sieving them through a 2 mm mesh. Following sieving, the soil samples were divided into 3 parts, 2 of which were kept at −80 °C for microbial analysis and quantification of soil nitrification rate. The third portion was air-dried at room temperature for 48 h before being subjected to soil physicochemical analysis.

### 2.2. Soil Physicochemical Analysis

Soil organic matter was determined by the potassium dichromate method (external heating) [38], and total nitrogen (TN) was determined by the semi-micro Kjeldahl method [39]. Soil pH was determined using a glass electrode at a soil-to-water ratio of (1:2.5) [40]. The alkaline hydrolysis diffusion method was used to quantify the available nitrogen (AN) [41]. The pH of the soil was determined using distilled water extracts and a pH meter [42]. A platinum electrode was used to measure the soil’s electrical conductivity (EC) in a water–soil suspension (5:1) [39]. An Auto Analyzer 3 (Bran+ Luebbe GmbH, Germany) was used to determine ammonium nitrogen (NH_4_**^+^**-N) and nitrate–nitrogen (NO_3_**^−^**-N) [43,44]. The colorimetric Mo-Sb method was used to quantify soil phosphorus (TP) and available phosphorus (AP) [39].

### 2.3. Soil Genomic DNA Extraction and Determination of Quality and Quantity

FastDNA^®^ SPIN Kit for Soil (MP Biomedicals LLC, Solon, OH, USA) was used to extract total genome DNA from samples according to the manufacturer’s instructions. A Qubit 3.0 Fluorometer (Invitrogen, Carlsbad, CA, USA) was used to measure DNA concentration, and 0.8% agarose gel was used to verify DNA quality. For sequencing and qPCR, the samples were stored frozen at −80 °C.

### 2.4. ITS1 Gene Amplification, Purification, Library Construction and Sequencing

Next-generation sequencing, library preparations and Illumina MiSeq sequencing were conducted at GENEWIZ Inc. (Suzhou, China). Briefly, the fungal internal transcribed spacer 1 (ITS1) region was analyzed using the ITS1F and ITS2 primers [45]. The reaction mixture for polymerase chain reaction (PCR) consisted of 25 μL reaction mixture containing 2.5 μL of TransStart Buffer, 2 μL of dNTPs, 1 μL of each primer, and 20 ng of template DNA and ddH_2_O to the total volume 25 μL for each sample. The thermal cycle conditions for PCR were initially denaturated at 94 °C for 5 min, followed by 25 cycles of 94 °C for 30 s, 57 °C for 30 s, and 72 °C for 30 s and final extension at 72 °C for 5 min. The PCR product was checked on 1.5% agarose gel electrophoresis. The primers included adaptor sequences in addition to the ITS1 target-specific sequence for high-complexity library amplification and downstream NGS sequencing on the Illumina MiSeq. DNA libraries concentration was validated by Qubit 3.0 Fluorometer (Invitrogen, Carlsbad, CA, USA) and real-time PCR (Applied Biosystems, Carlsbad, CA, USA). According to the manufacturer’s recommendations, DNA libraries were multiplexed and added to an Illumina MiSeq instrument. (Illumina, San Diego, CA, USA). The sequencing was carried out using a 300 bp paired-end (PE) setup. The MiSeq Control Software (MCS) on the MiSeq instrument was used to perform image analysis and base calling.

### 2.5. Processing of Fungal Sequencing Data and Diversity Analysis

The forward and reverse readings were merged, and samples were assigned to their barcode. The barcode and primer sequences were cut using Cutadapt (1.9.1) [46]. Sequences that did not fulfill the quality filtering requirements of 200 bp, no ambiguous bases, and a mean quality score of >20 were removed. After that, the sequences were compared to the Ribosomal Database Project (RDP V 2.2) using the UCHIME method to look for chimeric sequences, which were subsequently eliminated. For the final analysis, the most successful sequences were used. Using the clustering program VSEARCH (1.9.6) against the UNITE ITS database (https://unite.ut.ee/ accessed on 18 May 2021) the sequences were grouped into operational taxonomic units (OTUs) at 97% sequence similarity. All OTUs were assigned a taxonomic category with a confidence threshold of 0.8 using the Ribosomal Database Program (RDP) classifier. The UNITE ITS database was used by the RDP classifier, which predicts taxonomic classifications from kingdom to species level. Alpha diversity analyses, namely Chao1, Shannon, Simpson, and ACE were calculated using QIIME (Version 1.9.1). Phylogenetic alpha diversity indices PD (Faith’s phylogenetic diversity and SR (species richness)) were calculated using the ‘Picante’ package R [47]. Beta diversity analysis was performed using QIIME software (version 1.9.1) to assess differences in community complexity among samples and calculate UniFrac distance. Non-metric multi-dimensional scaling (NMDS) analysis based on Bray–Curtis distance was used to investigate composition differences among fungal communities in the R software.

### 2.6. Quantitative Real-Time Polymerase Chain Reaction (qPCR)

The 18S rRNA gene for the fungal community was quantified by quantitative polymerase chain reaction (qPCR). The 18S rRNA gene was quantified using the FR1/FF390 primers [48]. Absolute quantification was performed using the Applied Biosystems QuantStudioTM 6 Flex real-time PCR system (Life Technologies Corporation, Carls-bad, CA, USA). Hieff™ qPCR SYBR^®^ Green Master Mix (Yeasen, Shanghai, China) was used as a qPCR amplifier. The total volume of the qPCR reaction was 20 μL, with 10 μL 2 × SYBR^®^ Green Mix, 0.4 μL forward and reverse primer (10 mol/μL), 1 μL diluted (10 ng/μL) DNA template, and 7.4 μL double distilled water. The qPCR reaction protocol was based on Siles and Margesin’s description [49], with the following minor changes: pre-denaturation at 95 °C for 5 min, followed by 40 cycles at 95 °C for 15 s, annealing at 55 °C for 30 s, and extension at 72 °C for 60 s.

### 2.7. Null Model Based Phylogenetic Diversity Matrices and Ecological Assembly Analysis

To evaluate the phylogenetic diversity and ecological assembly processes, we utilized the null model “taxa. labels” to quantify the phylogenetic alpha diversity, such as nearest taxa index (NTI), which is a measure of mean pairwise phylogenetic distance at the local level and quantifies tip-level divergences (putting more emphasis on terminal clades and is akin to “local” clustering) in phylogeny. Briefly, this method records original phylogenetic distances in a phylogenetic tree and then generates 1000 randomizations of the phylogenetic tree (whilst keeping richness preserved) to calculate the phylogenetic distances on these distributions. Afterward, the mean and standard deviation of these distances obtained from a randomization procedure was used in a method called “statistical effect size” for comparison against the original distances to give NRI/NTI estimates. The values of NRI/NTI hold importance as they can be used to discern an underlying ecological mechanism. For a single community, NTI values > +2 suggest environmental filtering (phylogenetic clustering), and values < −2 indicate competitive exclusion (phylogenetic overdispersion) among species as the driver of community structure [50]. Although both NRI and NTI use similar thresholding cut off, NTI is typically preferred over NRI for ecological interpretation because of the presence of phylogenetic signals across short phylogenetic distances [51]. Therefore, in the current study, we calculated NTI. We quantified mean-nearest-taxon-distance (MNTD) and the nearest-taxon-index (NTI), using ‘mntd’ and ‘ses.mntd’ functions in R package ‘picante’ [52].

Ecological null modeling was performed in order to investigate the ecological drivers controlling fungal communities. Specifically, β-nearest taxon index (βNTI) and the Bray–Curtis-based Raup–Crick metric (RCbray) were used to determine contributions from selective and dispersal-based processes, respectively. First, β-mean nearest taxon distance (βMNTD) was calculated for each possible pairwise comparison within fungal communities using ‘comdistnt’ in R package “picante” [52]. By comparing these observed βMNTD values to those obtained from 999 community randomizations, βNTI was calculated according to Stegen et al. [53,54]. These βNTI results can then be used to investigate the phylogenetic turnover within the community and to understand whether deterministic (i.e., selection) or stochastic (i.e., random) processes affect community composition. If a |βNTI| value exceeds 2, a deterministic process shapes the fungal community; if a βNTI value is less than 2 and greater than −2, a stochastic process affects the community. Deterministic processes can then be distinguished based upon the sign of the βNTI value. When βNTI is greater than 2, communities are significantly more different than would be explained by random chance due to variable selection. If βNTI is less than −2, communities are significantly more similar than would be expected by random chance due to homogeneous selection [55]. In addition to βNTI calculations, the RCbray was used to further distinguish observed stochastic processes as described by Stegen et al. and chase et al. [54,56] on pairwise comparisons with |βNTI| < 2. Briefly, the RCbray index is the magnitude of deviation between observed and expected Bray–Curtis values, varied from −1 to 1, and we compared the values of |βNTI| < 2 and (RCb_ray_). In the context of |βNTI| < 2, a value of (RC_bray_) > +0.95 indicates that the observed turnover is dominated by the dispersal limitation, (RC_bray_) value < −0.95 indicates the effect of homogeneous dispersal, and (RC_bray_) < 0.95 value indicates the influence of drift. The undominated concept described a situation in which ecological drift, rather than dispersion or selection, was the fundamental source of differences in population compositions (stochastic birth and death events cause population size to fluctuate) [53].

### 2.8. Evidence of Neutral and Niche Processes by Sloan’s Neutral Community Model for Fungal Community

The neutral community model (NCM) is a neutral-based process analysis that has been effectively applied to various ecological approaches. NCM is an effective technique for evaluating stochastic processes affecting community assemblages [57,58]. To determine the importance of neutral processes in the assembly of fungal taxa (OTUs in our data), we applied Sloan’s neutral community fit model [35,59]. Sloan’s model was fit to the relative abundance of OTUs observed in all samples (metacommunity) and their observed detection frequency in each sample. We used R package MicECO with the neutral function fit according to Burns et al., [58]. In the current study, we first fit Sloan’s model to the overall fungal community in all samples then we applied this approach to each soil layer to determine the role of neutral processes on overall community assembly, then at a specific soil layer. In general, the parameter m indicates an estimate of the migration rate reveals the probability that a dead member would be replaced by immigrants from the metacommunity, which disperse into the local community [60]. The calculation for the best fit of the rate of m was estimated by using nonlinear least squares and maximum likelihood methods. Sloan’s neutral model categorizes OTUs in the population according to whether they occur more often than ("above" partition), within ("neutral" partition), or less often than ("below" partition) the 95 percent confidence interval. Briefly, the OTUs above the partition were vigorously selected for, while the OTUs below the partition were forcefully selected against. The goodness-of-fit of the Sloan neutral community model was assessed using the generalized R-squared (Rsqr = 1– the sum of squares of residuals/the total sum of squares) [61]. A high R^2^ value (> 0.2) shows that a neutral process of ecological drift and dispersal contributes more to community assembly, whereas a low R^2^ value (0.2) indicates that poor fitting occurs and other mechanisms (e.g., selective growth/attachment) contribute to community assembly [62,63]. The log-normal model (LNM), based on niche theory were used to quantified the contribution of niche processes [64]. The model assumes that the logarithmic form of species abundance is normally distributed. The model is defined as:

$$S\left(R\right)=S0e-\left(\alpha 2R2\right)$$

where S_(R)_ is the number of species within the Rth octave to the left and right of the symmetrical curve; S_0_ is the number of species within the modal abundance octave and 1/*a* is the distribution width [65]. The log-normal model was performed using the “radfit” function in the vegan package in [66]. Akaike information criterion (AIC) values were used to test for significant differences between the expected and observed species abundance distribution patterns. The AIC values close to zero indicating best model fitting. The AIC calculations were carried out in R using the package ‘vegan’ [67].

### 2.9. Statistical Analyses

We used Pearson correlation analyses to investigate the relationship between environmental factors. One-way analysis of variance (ANOVA) was used to compare gene copy numbers between soil layers and altitudes. Furthermore, Two-way analysis of variance (ANOVA) was used to analyze the effect of soil layers and altitudinal gradients on fungal gene abundance. Pearson correlations (r) were also run among soil physiochemical properties and fungal abundance data. The influence of physical and chemical factors on the distribution of fungal communities was investigated using canonical correspondence analysis (CCA). A variance portioning analysis (VPA) was used to assess the impact of individual environmental factors and geographic distance on the composition of fungal communities. The Mantel test was used to show the correlation between the physicochemical properties of the samples and the community structures of the most dominant fungal genera, and the results were combined with the “ggClusterNet” package in R [68]. The correlation analysis of beta diversity indices such as taxonomic (Bray–Curtis), phylogenetic (weighted UniFrac), null model-based beta diversity (βMNTD), and βNTI values (stochastic/deterministic processes) with environmental and geographic distance were performed by Mantel Pearson correlation analysis. The ‘ade4’ package in R was used to calculate significance using 9999 permutations [69]. Using standardized Euclidean distances, total geographic, and environmental differences across samples were estimated. Using partial Mantel tests with Pearson’s correlation coefficient and 999 permutations, the correlations between βNTI and the 2 geographical variables (Euclidean distances in environmental parameters and geographic distance) were investigated.

## *3.* Results

### 3.1. Soil Physicochemical Analysis

Nine soil physicochemical parameters were measured (Figure S1). The pH values were higher in the surface soil layer at lower altitudes while, on the contrary, pH values decreased with the increase in altitude. Soil EC gradually increased along with soil depth and decreased along with altitudinal gradients. The highest electrical conductivity was found in a deeper layer at 1789 m altitude, which was 130.67 μs/cm while the lowest values were noted at 3837 m. Soil available nutrients, including SOM, decreased along with soil depth and increased along with altitude. Soil TN and AN increased along with soil depth and altitudinal gradients. We found a decline in NH_4_^+^-N from surface to deeper soil layers, and NH_4_^+^-N increased gradually along with elevation in the surface soil layer. Asymmetric variations are found in the availability of NO_3_^−^-N along with elevation and soil layers. Soil chemical elements TP showed asymmetric distribution along with elevation gradient while TP concentration increased along with soil depths and AP increased along with soil layers and elevations. Pearson correlation analysis (Table S2) showed that pH was significantly positively correlated with EC while negatively associated with SOM, TN, AN, NH_4_^+^-N, TP, and AP concentration in soil. The soil pH values negatively correlated with the availability of nutrients in the soil. Soil electrical conductivity negatively correlated with SOM and positively correlated with NO_3_^−^-N, which indicated accumulation of soil organic matters significantly alters soil conductivity and pH values in natural soil habitat. Soil organic matters positively correlated with TN, AN, AP, NH_4_^+^-N, and TP. The nitrogen availability, either organic nitrogen or alkaline nitrogen, showed similar trends as SOM.

### 3.2. Quantification of Fungal Gene Abundance

**Figure 2:**
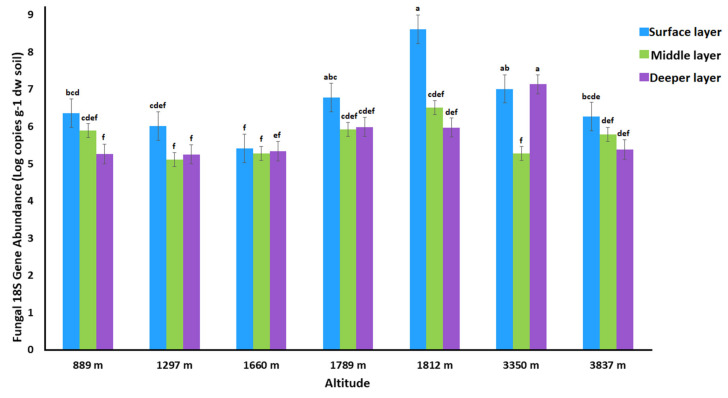
The quantitative gene abundance per gram dry soil in different soil layers and altitudes. Different lower-case superscript letters on different bars indicate significant differences between samples (Duncan’s test, *p* < 0.05).

The fluorescent quantitative polymerase chain reaction (qPCR) was used to determine the amount of 18S rRNA fungal gene copies per gram of dry soil across soil layers and an altitudinal gradient (Figure 2). The reaction was performed in triplicate for each sample, and mean values were considered for quantification. The 18S rRNA gene log copy numbers in all samples ranged from 5.118 to 8.610 per gram of dry soil. Considering soil depth, the fungal gene copy numbers were the highest in the surface soil layer for all altitudes except 3350 m, where a slightly higher value was recorded for the deeper layer while the middle soil layer exhibited significantly lower gene copies. Regarding altitudinal gradients, we found the lowest gene copies at lower altitudes, and gene abundance increased with elevational gradients, while the highest gene copies were observed at altitudes of 1812 m (8.610 log copies per gram of dry soil). Two-way ANOVA results shown in (Table 1) indicating that altitudes and altitudes with soil depth effect strongly (F = 5.88 and 7.74 *p* = 0.000) fungal gene abundance than soil depth alone (F = 5.81, *p* = 0.006). Interestingly, the 3350 m sample middle layer had a significantly lower value. For samples, 889 m, 1812 m, and 3837 m, the gene abundance decreased with the soil depth, while for 1297 m, 1660 m, and 1789 m samples, slightly higher results were obtained for the deep layer than middle layers. Furthermore, we investigated the correlation between environmental variables and fungal gene abundance. Fungal gene abundance surprisingly showed a non-significant correlation with soil pH, EC, and SOM, while it was positively correlated with TN, AN, NH_4_^+^-N and AP (Table S2).

### 3.3. Sequence Analysis and Fungal Community Composition

Illumina yielded valid 4,208,571 sequences by amplifying the ITS1 region from 63 soil samples with an average number of 66802 (SD = 20,052.61) sequences per sample. After quality checking and removing reads present in one replicate, a total of 41,769,64 high-quality reads were obtained with an average number of 66,301 (SD = 19,716.66) reads per sample and selected for further analysis. The sequence data were assigned to operation taxonomic units (OTUs) based on a 97% similarity level; 8039 fungal OTUs were classified. The annotation of assigned OTUs revealed almost 82% of the fungal sequence being classified at the phyla level. A total of 19 phyla, 58 classes, 134 orders, 299 families, and 702 genera were identified. The fungal phylum Ascomycota was dominant (48%), followed by Basidiomycota (14%) and Mortierellomycota (12%). The fungal phylum Ascomycota richness decreased from surface to deep soil layers (from 71%, 56%, to 37%, respectively). The overall asymmetric richness of Ascomycota, Basidiomycota, and Mortierellomycota along altitudinal gradients was found, the highest richness of Ascomycota, Basidiomycota, and Mortierellomycota was found at altitudes 1789, 3350, and 3837, respectively, while the lowest richness was found at altitudes 3350, 889, and 1297, respectively (Figure S2). Regarding soil layers, the dominance of fungal phylum, Ascomycota decreased sharply from surface to middle soil layer while it slightly increased in deeper soil layer (from 59.88% to 42.61% and 43.61%, respectively). Fungal phylum Basidiomycota decreased from the surface to middle soil layer and increased in the deeper soil layer, while phylum Mortierellomycota increased significantly from surface to deeper soil layers (from 3.97% to 16.31%). We also analyzed the fungal community composition at the genus level. A total of 702 fungal genera were identified from all samples; the top 15 genera are shown in (Figure S3). Unclassified fungal taxa at the genus level accounted for 33.57%, followed by Mortierella, Fusarium, Endocarpon, and f_unidentified_Unclassified (9.51%, 7.57%, 4.32%, and 4.01%, respectively). The classified genus Mortierella dominated at altitudes 889 and 1669 (14.15% and 14.26%, respectively), decreasing sharply at the highest altitudes (4.34%). The fungal genera Solicoccozyma and f_Mortierellaceae_Unclassified increased along the altitudinal gradient (from 0 to 7.27% and 6.28%, respectively). With respect to soil layers, the classified fungal genera Mortierella, Fusarium, Solicoccozyma, Lactifluus and f_unidentified_Unclassified, f_Nectriaceae_Unclassifi, and f_Mortierel also increased along soil layers. The relative abundance of genus Endocarpon decreased sharply from surface to deeper soil layers (from 12.85% to 0%).

### 3.4. Alpha Diversity Analysis

The fungal richness index (ACE and Chao1 estimator), diversity index (Shannon, Simpson index), phylogenetic diversity index (PD), and species richness index (SR) were all calculated (Table S3). Our results indicated that the indices SR, ACE, and Chao1 values decreased from surface to deeper soil layers (from 563, 645, and 646 to 207, 219, and 221, respectively), while fungal species richness (SR) increased with the increase in altitudinal gradients (from 205 to 573). Furthermore, fungal richness increased along with altitude, highest richness was found at 3887 m in the surface soil layer. The diversity index Shannon and Faith’s phylogenetic diversity (PD) values showed similar diversity patterns; fungal diversity increased along the altitudinal gradient (from 3.28 to 6.26 and 58.71 to 143.10, respectively), while it decreased among soil layers (from 5.13 to 4.16 and 126.30 to 62.10, respectively). Our findings indicated that the fungal community diversification rate was higher at higher altitudinal gradients and reduced along with soil depth. Across all altitudinal gradients and soil layers, we calculated NTI from standardized size effect of mean nearest taxon index using the null model were positive and range between 2 and −2 (Figure S4A). In addition, NTI values were relatively higher at surface soil layers than middle and deeper soil layers, while NTI values increased significantly along with altitudinal gradients (Figure S4B). Analysis of taxonomic and phylogenetic beta diversity

**Figure 3:**
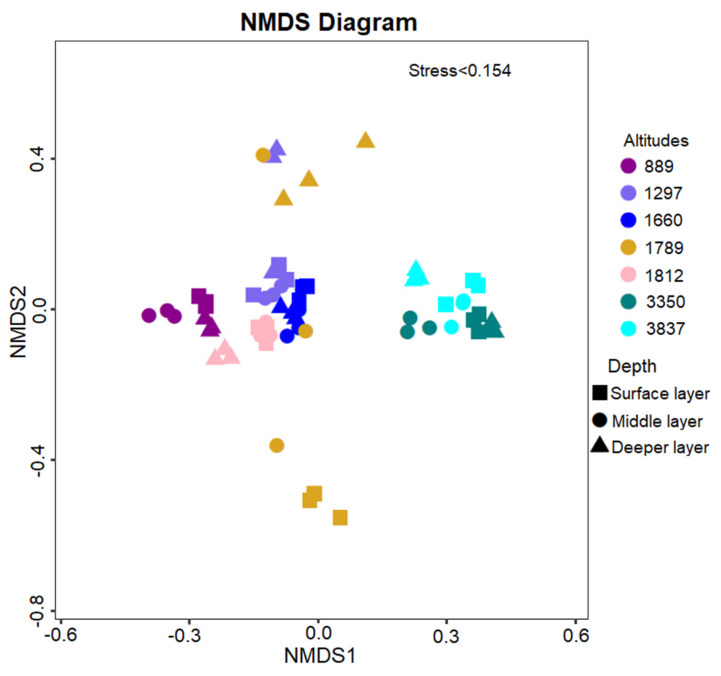
Fungal Community Bray–Curtis distance-based Non-Metric Multi-Dimensional Scaling plot at OTU level of soil samples among different soil layers and altitudinal gradients.

The distribution of the fungal community was distinct among lower and higher altitudinal gradients based on taxonomic weighted Bray–Curtis based NMDS analysis (Figure 3). The fungal community with closer altitudes showed lower dissimilarity. Similarly, the composition of the fungal community in altitudes 3350 and 3837 showed more similarity for distinct community composition than lower altitudes. The community dissimilarity between soil layers was not significantly different, while at middle altitude, 1789 in the community in a deeper soil layer were distinct from the surface and middle soil layers. For further confirmation, we applied taxonomic Bray–Curtis dissimilarity, phylogenetic weighted UniFrac, and βMNTD analysis to investigate the role of altitudinal and/or soil layer on community distribution. Linear regression analysis of beta diversity indices, Bray–Curtis dissimilarity, w.Unifrac, and βMNTD (Figure S5), showed that fungal community dissimilarity along altitudinal gradients was very low (r^2^ = 0.008, r^2^ = 0.005, r^2^ = 0.0002, respectively). On the other hand, fungal dissimilarity along with soil layers was also low; comparatively, fungal communities present in the middle soil layer were much more diverse than the surface and deeper soil layers. Fungal community similarity was higher in deeper soil layers. These results suggest that the composition of the fungal community in the middle soil layer is influenced by altitudinal gradients compared to the surface soil and deeper soil layers (Figure S6).

### 3.5. Quantification of Neutral Processes and Migration Rate

**Figure 4:**
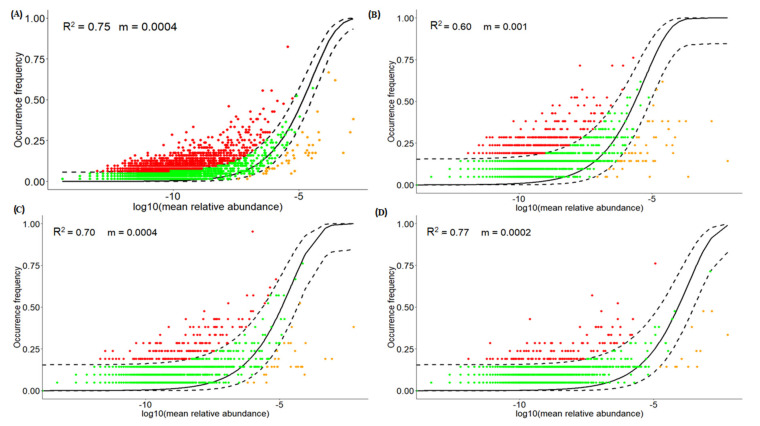
Fit of occurrences frequency of different fungal OTUs as a function mean relative abundance applying Sloan’s model [35]. Red and yellow dots indicate more or less frequently than given by the model. Green dots between dashed lines indicate 95% confidence intervals of the model. R^2^ denotes fitness of model, R^2^ > 0.2 fitness of model more, m indicates migration rate. (**A**) Overall community, (**B**) surface layer, (**C**) middle layer, (**D**) deeper layer.

We assumed that habitat heterogeneity between soil layers and altitudinal gradients might lead to major spatial differences in fungal community assembly. It is important to examine whether neutral and/or niche processes play a role in the shaping of microbial communities in different soil layers and throughout the altitudinal gradients. According to Sloan‘s neutral model, the neutral model presented an excellent fit to the distribution of the fungal community (R^2^ = 0.75). These findings suggested that there are more serious dispersal limits for the eukaryotic community (Figure 4). Dispersal limitation increased along with soil layers, and a lower migration rate was noted at deeper soil layers for the fungal community. Furthermore, we employed a log-normal model to validate the role of niche processes in the fungal community composition. Based on AIC values (7564.75), the results showed that the log-normal model is not fitted on all soil samples, which is constant with NTI values and indicates that neutral processes were the main drivers for fungal community assemblage (Figure S7).

### 3.6. Assembly Processes of the Fungal Community and Contribution of Deterministic and Stochastic Processes

**Figure 5:**
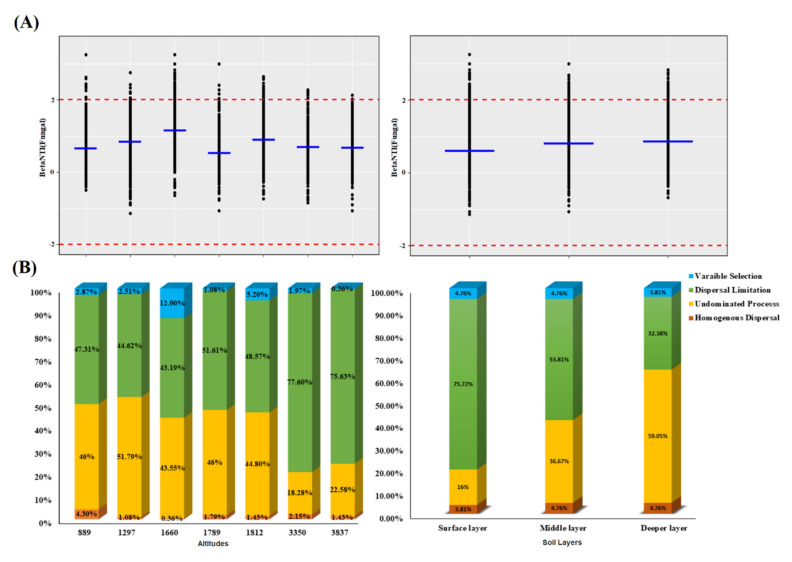
(**A**) Scatter plot of βNTI across spatial scales of Tibetan soil. Blue line indicating the median values of each sampling location. (**B**) Quantitative analysis of relative importance of each assembly process that govern the fungal community composition.

We estimated null model-based βNTI values to see if stochastic or deterministic mechanisms may better explain the assembly processes of fungal community composition across soil layers and altitudinal gradients. The distribution of βNTI across elevational gradients showed stochastic processes were dominant (96.15%), while the lowest stochastic rate was noted at altitude 1660. These findings indicated that the fungal community was assembled by the stochastic process with βNTI values between −2 and 2 across altitudinal gradients (Figure 5A). Among soil layers for the fungal community, the stochastic rate increased from surface to deeper soil layers (from 95.24% to 95.55%), suggesting that the fungal community present in the surface soil layer is slightly vulnerable to environmental selection. The (RCbray)-based results showed that the influence of selection (deterministic processes) on the fungal community structure along the altitudinal gradient was very low, while a contribution of 12.9% of variable selection was detected at altitude 1660 site. The dispersal limitations contributed a greater proportion to the fungal community along altitudinal gradients, while the dispersal limitations decreased from the surface to the middle and deeper soil layers (from 75.72% to 53.81 and 32.38%, respectively), followed by undominated processes. Furthermore, at each soil layer, the undominated process increased from surface to middle and deeper soil layers (from 16% to 36.67% and 59.05%, respectively) (Figure 5B). Hence, our findings indicated a contrary trend in dispersal limitation and undominated community assembly processes in controlling the composition of fungal communities.

### 3.7. Integration of Environmental and Spatial Distance in Shaping Fungal Communities

**Figure 6:**
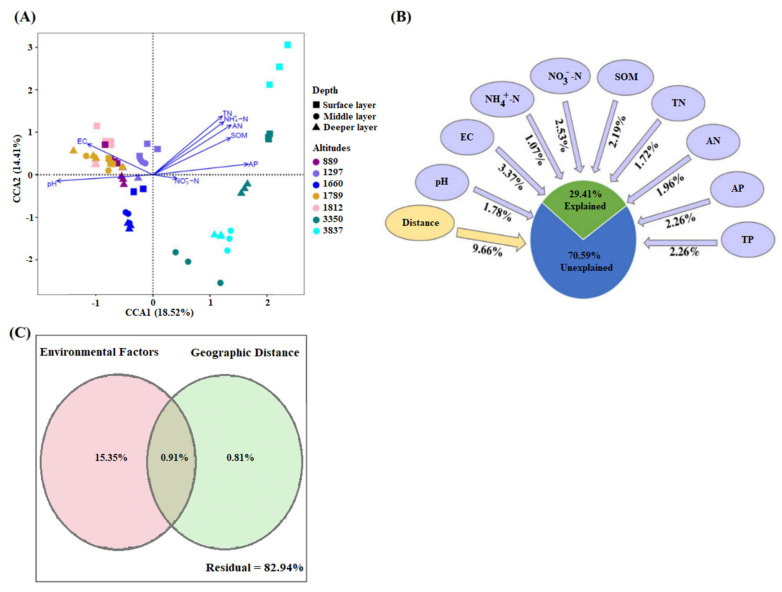
(**A**) Relationship between environmental variables and fungal community structures. The length of the arrow represents the degree of correlation between environmental variables and community structures. (**B**) Variance partitioning analysis of individual environmental variables and spatial distance of soil fungal communities. (**C**) Variance partitioning analysis of combined environmental factors and geographic distance. Numbers indicate the proportion of explained variation and residuals indicate unexplained variations.

To explore the key drivers shaping the fungal community structure and composition among soil layers along with altitudinal gradients, we utilized comprehensive statistical methods to investigate the role of environmental variables and/or spatial distance. To analyze the influence of environmental factors on the relative composition of fungal communities, canonical correspondence analysis (CCA) was performed. The results showed that 18.52% and 14.41% variations at OTUs levels were explained by the X and Y axis, respectively. There was a positive correlation between the EC and fungal community present in lower altitudes, while pH positively correlated with fungal community composition at altitude 1660. The fungal community at high altitudinal gradients 3350 and 3837 showed a negative correlation with EC and pH. These findings suggest EC and pH are major environmental variables that influence fungal community composition at lower altitudinal gradients (Figure 6A). CCA analysis could not explain the contribution of individual variables; therefore, we performed variance portioning analysis (VPA) analysis. VPA results showed that (29.40%) community variations were explained by environmental factors and geographic distance. The geographic distance and EC explained 9.65% and 3.36%, respectively (Figure 6B). The environmental and geographic distance showed the lowest proportion of explained variables. Environmental distance and geographic distance explained 15.35% and 0.8% variations, respectively, while a mutual effect of both parameters revealed (0.9%) variations in fungal community distributions (Figure 6C).

**Figure 7:**
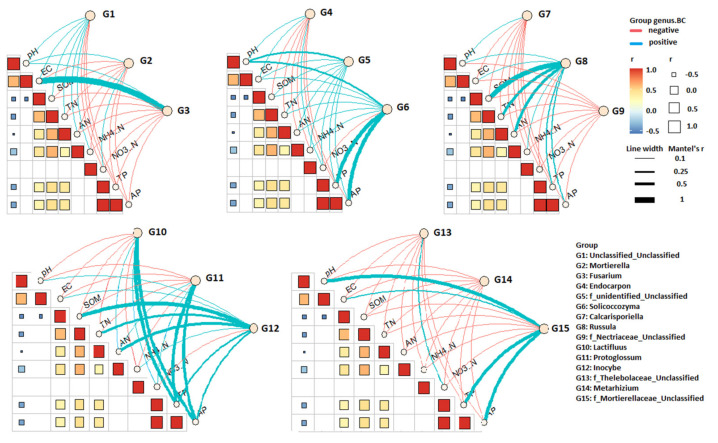
Pairwise comparisons of environmental factors and their effects on top dominated fungal genera community composition. Spearman’s correlation is shown in a color gradient. Fungal genera based on Bray–Curtis distance are correlated to environmental factors by Mantel test, with edge representing Mantel’s *r* for correlations and the color corresponding to the significance.

The relationship of dominant fungal genera based on Bray–Curtis distance with environmental factors was analyzed by the Mantel test (Figure 7), and the results indicated that TP and AP significantly influenced the compositions of *Russula*, *Inocybe*, *Lactifluus*, *Solicoccozyma*, *Protoglossum,* and *f_Mortierellaceae_Unclassified*. The nitrogen content (TN and AN) also influenced the composition of *Russula*, *Inocybe*, *Lactifluus*. SOM influenced the composition of *Russula* and *Inocybe*. Soil pH showed a positive correlation with *f_unidentified_Unclassified*, *Solicoccozyma,* and *f_Mortierellaceae_Unclassified*. These findings suggest that the composition of *Russula* and *Inocybe* were affected by multiple factors, including TP, AP, TN, AN, and SOM, while pH and EC indicate minor roles in the composition of dominant fungal genera.

**Figure 8:**
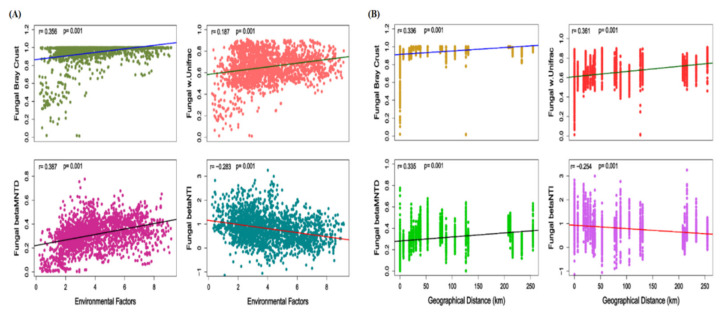
(**A**) Scatter plot diversity indices on Mantel test Spearman correlation “r” show the relationship of taxonomic, phylogenetic null model-based and βNTI values of fungal communities with environmental variables (Euclid based distance). (**B**) Scatter plot diversity indices on Mantel test Spearman correlation “r” show the relationship of taxonomic, phylogenetic null model-based, and βNTI values of fungal communities with geographic distance.

In order to examine whether the local environmental variables and/or spatial distance exhibited an influence on the beta diversity of fungal communities, we performed Mantel Spearman correlation analysis. Fungal taxonomic and phylogenetic beta diversity indices Bray–Curtis dissimilarity and weighted UniFrac distance and βMNTD showed a significant positive correlation with environmental variables. The βMNTD and Bray–Curtis dissimilarity index showed higher correlation with environmental factors than weighted UniFrac distance (r = 0.387 and r = 0.356, *p* = 0.001 respectively). While βNTI values showed a negative correlation with environmental variables, these findings are similar with RC_bray_ analysis, which showed only 4.4% influence of environmental variables. Among soil layers, both diversity indices showed that environmental variables exhibit the strongest correlation with the community present in the deeper soil layer (r = 0.91 and r = 0.92), indicating the highest influence of environmental variables on diversity (Figure 8A). Further, we investigated the role of geographic distance on diversification of the fungal community. Mantel test results showed the influence of geographic distance on the fungal community diversity indices (Figure 8B). The phylogenetic beta diversity weighted UniFrac distance showed relatively higher correlation (r = 0.361, *p* = 0.001) with geographic distance than taxonomic Bray–Curtis and βMNTD diversity index (r = 0.336 and 0.335, *p* = 0.001 respectively). The βNTI showed a negative correlation with geographic distance. Furthermore, the middle soil layer diversification rate and geographic distance showed a higher correlation while weak correlations were found in deeper soil layers (Table S4).

## 4. Discussion

High altitudinal soils are unique and may be challenging for life due to specific environmental factors such as their oligotrophic nature, dramatic freeze-thaw cycles, and exposure to radiations [70,71,72]. Several studies on soil fungi have been published at various elevations, including the Himalayan Mountains range in Nepal, the Colorado Rocky Mountains in the United States, the Milla and Segrilla Mountains in Tibet, central Veracruz in Mexico, Mt. Fuji in Japan, and Mt. Kinabalu in Malaysia [3,72,73,74,75,76]. In addition, some studies also reported fungal community composition and diversity along the altitudinal gradients but have mainly focused on the topsoil layer [77,78,79]. In this study, we conducted a comprehensive analysis of fungal abundance, composition, diversity, and assemblages between different soil layers and altitudinal gradients by qPCR analysis and ITS1 region Illumina sequencing. We found that the fungal gene log copies are in the range of 5.11 to 8.61 per gram of dry soil among all soil samples, which was higher than found by [49]. The fungal gene abundance dramatically increased from lower to higher altitudinal levels from 5.840 to 8.610 log number gene copies, respectively. Regarding soil layers, the surface layer had the higher fungal gene copy numbers, while the middle layer harbored the lowest gene copy numbers. These findings are good in line with previous studies reporting that the fungal gene abundance was positively associated with altitudinal gradients [80,81]. Previous studies have shown that change in fungal communities can be associated with soil texture, pH, and soil nitrogen availability [10,82,83,84]. Some studies also reported that microbial biomass of mountain ecosystems is affected by coniferous litter inputs on surface layers [85,86]. The decomposition and litter inputs lead to the accumulation of C, N, and EC in alpine and subalpine soils [49]. Previously, it was reported that environmental variables N, C, and EC were positively correlated with fungal abundance [87]. In this study, we found that fungal gene abundance was positively correlated with N and P, while soil organic matter and pH showed non-significant correlations. We can conclude that higher fungal gene abundance in the surface soil layer may be due to the higher availability of nitrogen and environmental conditions.

In this study, we found that the *Ascomycota* (*Leotiomyceta)* was dominated by fungal phyla followed by Basidiomycota (*Agaricomycetes*) and *Mortierellomycota.* Some differences in the relative abundance of these two phyla were observed in the different ecosystems, for example, compared with desert soils, forest soils had more Basidiomycota. However, our findings are in good agreement with the previous study reported on the Tibetan Plateau, such as Zhang et al. [88] who reported *Ascomycota* and *Basidiomycota* accounting for 83.6 % of fungal sequences in the Tibetan Plateau, while we found comparatively lower abundance 62% of fungal sequences. *Ascomycota* accounted for 48% fungal sequence. This might be due to their higher ability to decompose cellulose as compared to other fungal taxa [89]. At higher altitudinal levels, we found a relatively higher abundance of *Ascomycota*, which may reflect the specific ecological functions of the subalpine cool temperate zone’s ecosystem. A significant decline in *Ascomycota* was observed from surface to deeper soil layers (from 71% to 37%) because above-ground fungal community was closely related to vegetation, which indicated that above-ground plant vegetation could be an important factor that determined *Ascomycota* community composition. At genus level fungal taxa distributions, we found saprotrophic *Mortierella* and cosmopolitan *Fusarium* followed by lichen-forming *Endocarpon* fungal genera. We observed the asymmetric abundance pattern of *Mortierella* along altitudinal gradients, while other studies reported this to have increased in abundance along elevation [90]. We also found that *Mortierella* abundance showed a non-significant correlation with environmental variables; it may track changes in nutrient quality rather than quantity or be partially determined by vegetation turnover due to its potentially endophytic trophic lifestyle. In addition, a comprehensive ecological study of *Mortierella* at higher elevational gradients in relation to plant roots needs to be further investigated. The pathogenic cosmopolitan plant *Fusarium* was dominant in our study, as also reported in the soil studied by Devi et al. [91]. The fungal genus *Endocarpon* decreased sharply from surface to deeper soil layers. The reduction of *Endocarpon* along soil layers may be due to increases in soil moisture content and its ability to tolerate harsh conditions [88,92]. We found a stronger impact of environmental variables such as TP, AP, TN, AN, and SOM on fungal genera *Russula* and *Inocybe*. Our results were consistent with previous reports that *Russula* abundance in the soil ecosystem was associated with the C:N ratio [93,94]. Elevation is a complex and indirect gradient along which many environmental factors vary with altitudinal levels. Several published studies highlighted the effect of elevation on Ectomycorrhizal fungal distributions such as Jarvis et al., who found that at a genus level, only *Russula* and *Inocybe* displayed a significant pattern, increasing in occurrence with altitude [90,95]. Another study reported that Ectomycorrhizal fungal taxa were host specific such as *Russula* genotypic diversity have preference for Pine Forest on mountain regions [96]. In this study, we observed that -diversity, species richness, AC, Shannon, and phylogenetic diversity index increased significantly at higher altitudes, and our findings were contrary to previous research [97,98]. Our contrary findings may be due to different elevation gradients because other studies included lower altitudinal gradients (24 to 2600 m). Another possible reason could be the study site and the Tibetan Plateau exhibiting unique environmental conditions [99], such as several studies reporting that geographic location is also an important factor affecting microbial diversity [100]. As expected, NMDS showed that higher altitudes exhibit distinct fungal communities (Figure 3). The “local community” refers to a sample from one point showing fungal mean NTI values less than +2 indicating weak phylogenetic clustering. This provides evidence of the weak role of deterministic processes or environmental filtering at the local community level [47]. Several reasons can explain this phenomenon. Firstly, as elevation increases, environmental harshness can also increase factors affecting fungal diversity and richness [101]. Secondly, climate change significantly impacts soil fungal diversity along with elevation [102].

To measure the impact of geographical and environmental factors on fungal community composition, we conducted VPA analysis. Environmental factors and spatial distance played a limited impact on shaping the fungal community, according to the VPA analysis (29.40 percent). Several studies have found a significant proportion of unexplained variation in bacterioplankton and microeukaryote communities in different habitats and sampling areas [103,104,105]. Several reasons could explain our results. First, the unexplained variations in the fungal community in this study could be due to the exclusion of other important factors, such as temperature, radiation, and precipitation through VPA [106,107,108]. Second, several studies have found that microbial co-occurrence can alter community distribution, which is not measurable via VPA [109,110]. Third, the VPA has a tendency to underestimate the contribution explained by environmental factors [111]. This may also explain the low contributions to the microeucharyotic community variation by deterministic or selective processes. The Mantel test results indicated that beta-diversity indices Bray–Curtis dissimilarity, weighted UniFrac distance, βMNTD, and βNTI showed distinct trends along with spatial and environmental correlations. Our results clearly indicated that stochastic processes dominated (96%) over deterministic processes (|βNTI| < 2) in shaping the fungal community across altitudes and soil layers. Fungal βNTI showed a weak negative correlation with environmental variables and spatial distance, indicating less influence on fungal community assemblages. The negative correlation between fungal community and spatial distance and environmental distance indicates the importance of selection and drift [112,113]. To confirm the possible role of selection and drift, we performed the neutral community model (NCM).

Sloan’s [35] neutral model was used to determine the biogeographic distribution of fungal communities. The NCM findings suggest that the stochastic balance between fungal loss and gain (such as stochastic births, deaths, and immigration) played a role in the shaping of fungal communities across all locations. Furthermore, the role of stochastic processes was confirmed by a considerable distance-decay pattern of the fungal population among soil strata. Our results are similar to previous studies on microeukaryotic communities in subtropical rivers (R^2^ = 0.89) [114]. According to Hubbell’s neutral theory [19], the community similarity is predicted to decrease along with spatial (geographic distance) gradients due to dispersal limitations [115]. While these correlations are almost similar to previous studies on smaller spatial scales, such as planktonic and sedimentary bacterial communities (R^2^ = 0.775) [116], they are larger than coastal lakes in the Antarctic (R^2^ ≤ 0.50) [117]. The migration rate is an important indicator for neutral and non-neutral portioning. We found a lower migration rate in this study, which has been shown to indicate a higher dispersal limitation [114]. In recent studies, dispersal limitations have been widely accepted as a major driver for shaping fungal community assembly [118,119,120]. Further, we calculated βNTI values and performed RC_bray_ analysis to investigate the role of dispersal limitation and other ecological processes. While it was assumed in our study that higher environmental variability could lead to higher structure heterogeneity as reported in previous studies, but our results indicated that dispersal limitation and undominated processes dominated ecological processes shaping the fungal community. Some studies have reported that dispersion of fungi is higher [121], while other studies have suggested the larger size of fungi compared to other microbes [91]. In our study, we found higher dispersal limitations along altitudinal gradients, and dispersal limitations were at the topsoil layer, while undominated processes lead to fungal assembly in deeper soil layers. Previously, it was found that limited availability of nutrients can lead to structural variability in microbial communities [122,123]; the higher dispersal limitations along altitudinal gradient might be due to availability of limited nutrients along altitudinal gradients. These findings are in agreement with [124] who reported that fungal dispersal limitation in paddy soil decreased along with soil depth. The dispersion of fungi is still controversial, many studies have reported that fungi are free to disperse thus dispersal limitation does not exist [121], while other studies have reported that due to the large size of fungi over other microbes, their dispersal is limited [91].

## 5. Conclusions

This study revealed fungal community composition, diversity, and assembly processes along altitudinal gradients among soil layers using qPCR and high-throughput DNA sequencing techniques. A positive correlation between soil fungal gene abundance and altitude differences was found on the Tibetan Plateau. Our study shows that the composition and diversity of soil fungi at the surface are determined by elevation gradients. The plant pathogenic fungal genus *Fusarium* increased from surface to deeper soil layers and was positively correlated with EC, while fungal genera *Russula* and *Inocybe* showed a positive correlation with environmental variables. Soil physicochemical parameters explained only 15% of the variation in fungal community composition along the elevations. Our study clearly indicates that the fungal community is assembled by stochastic processes. However, dispersal limitations and undominated factors were major stochastic forces to drive community composition as βNTI analysis, NCM, and the log-normal model indicated the predominance of stochastic processes in community composition. Future studies on a larger scale with more environmental variables will provide insight into the factors that influence fungal communities in these unique environments. This study will help to better predict the responses of soil fungi to environmental changes at the landscape level.

## Figures and Tables

**Table 1:** The effect of elevations and soil layers on fungal gene abundance as indicated by two-way ANOVA.

Factors	Fungal Gene Abundance (Log Copies)	
	F	*p*
SL	5.813	0.006
A	5.881	0.000
SL × A	7.746	0.000

Values represent mean with standard error in parenthesis. SL, Soil Layers; A, Altitudes.

## Data Availability

Not applicable.

## Supplementary Materials

The following are available online at https://www.mdpi.com/article/10.3390/jof7121082/s1, Figure S1: Soil physiochemical factors distributions along with elevational gradients, Figure S2: The relative abundance of fungal phyla along with elevation gradients and soil layers. SL: Surface Layer, ML: Middle Layer, DL: Deeper Layer, Figure S3: The relative abundance of top 15 fungal genera along with elevation gradients and soil layers, Figure S4: (A) Boxplot of Nearest Taxon Index (NTI) values of fungal communities along altitudinal gradients and (B)soil layers, Figure S5: Linear regression analysis of fungal taxonomic and phylogenetic beta diversity indices among altitudinal gradients, Figure S6: Linear regression analysis of fungal taxonomic and phylogenetic beta diversity indices among soil layers and altitudinal gradients, Figure S7: The rank abundance distribution lognormal model based on Akaike Information Criterion (AIC). The letters above boxes indicate significant differences between samples (Duncan’s test, *p* < 0.05). Table S1: Characteristics of the study sites on the Qinghai-Tibet Plateau, Table S2: Pearson correlation analysis among soil physio chemical parameters and soil layers along with elevation gradients, Table S3: Fungal community alpha diversity analysis performed by One-Way ANOVA, Table S4: The Mantel test results showing relationship between fungal diversity indices and environmental and geographical distance for all pairwise samples using mantel test.
