# Supplementary material for: Distribution and Assembly Processes of Soil Fungal Communities along an Altitudinal Gradient in Tibetan Plateau
Source: J Fungi (Basel). 2021 Dec 16;7(12):1082. doi: 10.3390/jof7121082 (PMC8706254; doi:10.3390/jof7121082)
Supplement: Supplementary file 1 [file jof-07-01082-s001.zip › jof-1463629-supplementary.pdf]

Journal of Fungi

MDPI

Supplementary

Table S1. Characteristics of the study sites on the Qinghai-Tibet Plateau.

Altitude/m	Longitude	Latitude	Slope	Soil Type	Vegetation Types
889	98°52′4''E	26°12'39''N	Yes	Brown soil	Shrubs, herbs
1297	98°50′1''E	27°23'7''N	Yes	Brown soil	Trees, shrubs, herbs
1660	98°30′2''E	28°7′20''N	Yes	Brown soil	Trees, shrubs, herbs
1789	98°27′4''E	28°28′22''N	Yes	Calcareous cinnamon soil	Trees, shrubs, herbs
1812	98°29′2''E	28°16′50''N	Yes	Calcareous cinnamon soil	Trees, shrubs, herbs
3350	98°48′40''E	28°6′33''N	Yes	Calcareous cinnamon soil	Trees, shrubs, herbs
3837	98°45′29''E	28°4'29'N	Yes	Frozen embryonic soil	Trees, shrubs, herbs

Table S2. Pearson correlation analysis among soil physio chemical parameters and soil layers along with elevation gradients.

	pH	EC	SOM	TN	AN	NH_4^+-N	NO_3^--N	TP	AP	18s Gene Abundance
pH	1									
EC	0.602**	1								
SOM	-0.496**	-0.522**	1							
TN	-0.445**	−0.118	0.593**	1						
AN	-0.572**	−0.178	0.372**	0.615**	1					
NH_4^+-N	-0.275*	0.085	0.422**	0.614**	0.283*	1				
NO_3^--N	0.065	0.249*	−0.045	0−0.152	−0.081	0.236	1			
TP	-0.404**	−0.248	0.295*	0.463**	0.399**	0.065	−0.152	1		
AP	-.0584**	−0.239	0.311*	0.318*	0.426**	0.512**	0.363**	0.324**	1	
18s gene abundance	−0.084	0.196	0.005	0.413**	0.308*	0.547**	−0.008	0.109	0.387**	1

Pearson correlation analysis among soil samples. Note: EC, Election Conductivity; SOM, Soil Organic Matter; TN, Total Nitrogen; AN, Alkaline Nitrogen; TP, Total Phosphorus, AP, Available Phosphorus. (* $P < 0.05$, ** $P < 0.001$, $n= 6$).

Table S3. Fungal community alpha diversity analysis performed by One-Way ANOVA.

	The Diversity Index					
**Samples**	**SR**	**ACE**	**CHAO1**	**Shannon**	**Simpson**	**PD**
SL889	161 ± 55.139^{cA}	173 ± 48.72^{cA}	176 ± 48.30^{cA}	4 ± 0.38^{bA}	1 ± 0.08b^{A}	47 ± 14.29^{cA}
ML889	208 ± 29.194^{bA}	238 ± 38.08^{bA}	236 ± 38.42b^{A}	2 ± 0.37^{cB}	0.42 ± 0.11^{cB}	51 ± 5.95^{cA}
DL889	248 ± 129.44^{abA}	255 ± 133.96^{abcA}	256 ± 137.77^{abcA}	4 ± 0.4^{abA}	1 ± 0.03^{aA}	78 ± 42.79^{abA}
SL1297	620 ± 320.32^{bA}	681 ± 377.18^{bA}	684 ± 370.27^{bA}	6 ± 1.39^{aAB}	1 ± 0.16^{abA}	135 ± 40.47^{bA}
ML1297	415 ± 144.60^{abAB}	432 ± 145.72^{abAB}	447 ± 166.39^{abAB}	7 ± 0.34^{aA}	1 ± 0.001^{aA}	122 ± 28.16^{aA}
DL1297	128 ± 158.04^{bcB}	132 ± 164.01^{bcB}	134 ± 168.45^{bcB}	2 ± 2.98^{cB}	0.3 ± 0.5^{bA}	35 ± 37.18^{bcB}
SL1660	929 ± 131.03^{aA}	1094 ± 151.28^{aA}	1092 ± 150.07^{aA}	7 ± 1.29^{aA}	1 ± 0.09^{aA}	167 ± 17.55^{bA}
ML1660	172 ± 33.83^{bB}	179 ± 31.36^{bB}	182 ± 28.39^{bB}	5 ± 0.83^{abA}	1 ± 0.09^{abA}	57 ± 10.17^{cB}
DL1660	244 ± 46.03^{abB}	254 ± 42.53^{abcB}	255 ± 46.34^{abcB}	6 ± 0.51^{aA}	1 ± 0.01^{aA}	69 ± 8.08^{abB}
SL1789	147 ± 38.89^{cA}	171 ± 31.89^{cA}	176 ± 25.28^{cA}	1 ± 0.81^{cA}	0.32 ± 0.2^{cA}	39 ± 11.52^{cA}
ML1789	204 ± 224.29^{bA}	209 ± 222.79^{bA}	209 ± 223.92^{bA}	3 ± 2.77^{bcA}	1 ± 0.5^{bcA}	64 ± 56.06^{bcA}
DL1789	78 ± 18.34^{cA}	86 ± 28.68^{cA}	85 ± 28.19^{cA}	3 ± 0.73^{bcA}	1 ± 0.15^{abA}	17 ± 1.53^{cA}
SL1812	577 ± 10.97^{bA}	670 ± 27.67^{bA}	669 ± 30.03^{bA}	6 ± 0.32^{aA}	1 ± 0.01^{aA}	136 ± 1.82^{bA}
ML1812	561 ± 56.15^{aA}	612 ± 66.18^{aA}	615 ± 66.35^{aA}	6 ± 0.17^{aA}	1 ± 0.004^{abA}	139 ± 5.59^{aA}
DL1812	151 ± 45.39^{bcB}	157 ± 47.04^{bcB}	155 ± 45.96^{bcB}	4 ± 0.24^{abB}	8.87 ± 0.01^{bB}	65 ± 25.52^{abB}
SL3350	554 ± 14.47^{bA}	618 ± 35.57^{bA}	616 ± 39.74^{bA}	6 ± 0.08^{aA}	1 ± 0.01^{aA}	147 ± 7.97^{bA}
ML3350	499 ± 197.24^{aA}	526 ± 213.42^{aAB}	527 ± 213.82^{aAB}	6 ± 0.36^{aA}	1 ± 0.01^{abA}	117 ± 38.71^{abAB}
DL3350	250 ± 48.91^{abB}	290 ± 47.88^{abB}	288 ± 53.20^{abB}	3 ± 0.64^{bcB}	1 ± 0.08^{aB}	72 ± 10.09^{abB}
SL3837	958 ± 76.89^{aA}	1115 ± 74.09^{aA}	1113 ± 69.48^{aA}	7 ± 0.33^{aA}	1 ± 0.01^{aA}	212 ± 6.23^{aA}
ML3837	406 ± 143.51^{abB}	422 ± 151.84^{abB}	430 ± 159.59^{abB}	6 ± 0.65^{aB}	1 ± 0.04^{abA}	115 ± 24.21^{abB}
DL3837	354 ± 53.68^{aB}	366 ± 58.31^{aB}	375 ± 61.60^{aB}	6 ± 0.08^{aB}	1 ± 0.004^{aA}	103 ± 3.94^{aB}

SL: Surface Layer, ML: Middle Layer, DL: Deeper Layer, OTU: Operational Taxonomic Units, ACE: Abundance-based Coverage Estimator. Note: Values are means ± standard deviation (n =3), and the superscripts of different upper-case letters the same column indicated significant difference among soil layers at each altitude while superscripts of different small letter denoted significant difference among all altitudes for each soil layer (Duncan's test, $P < 0.05$) among soil samples.

Table S4. The Mantel test results showing relationship between fungal diversity indices and environmental and geographical distance for all pairwise samples using mantel test.

**Variables**		**Surface layer**		**Middle layer**		**Deeper layers**	
		r	*p*	*r*	*p*	*r*	*p*
	Bray-Crust	0.542	0.001	0.405	0.001	0.547	0.001
E. F	w.UniFrac	0.235	0.001	0.234	0.001	0.407	0.001
	βMNTD	0.51	0.001	0.572	0.001	0.478	0.001
	βNTI	−0.345	0.001	−0.196	0.001	−0.341	0.001
	Bray-Crust	0.395	0.001	0.386	0.001	0.275	0.001
G. D	w.UniFrac	0.443	0.001	0.646	0.001	0.199	0.001
	βMNTD	0.425	0.001	0.324	0.001	0.244	0.001
	βNTI	−0.291	0.001	−0.328	0.001	−0.273	0.001

"*r*" shows the degree of relationship, n =21. E.F; Environmental Factors, G.D; Geographic distance.

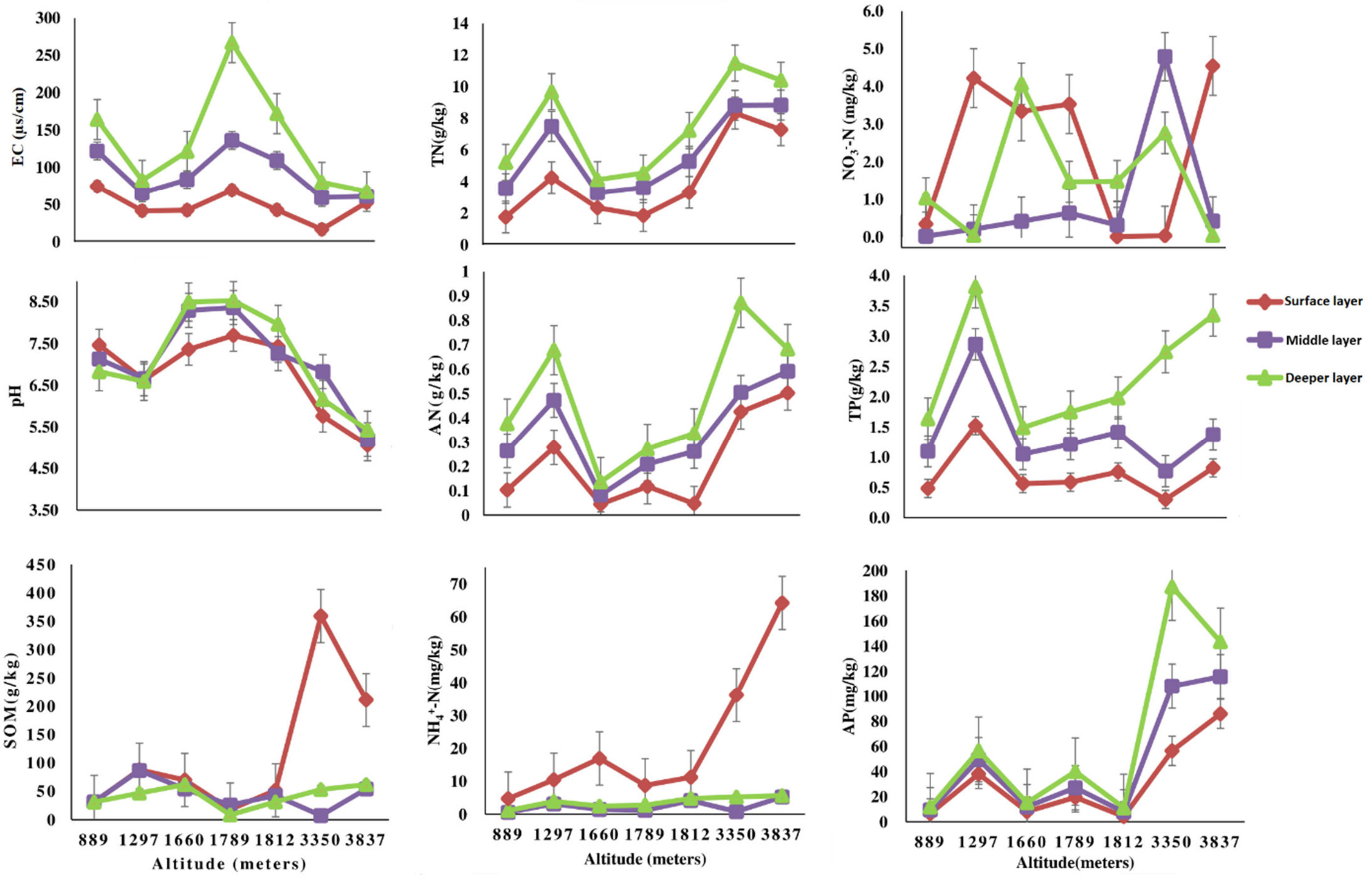

Figure S1. Soil physiochemical factors distributions along with elevational gradients.

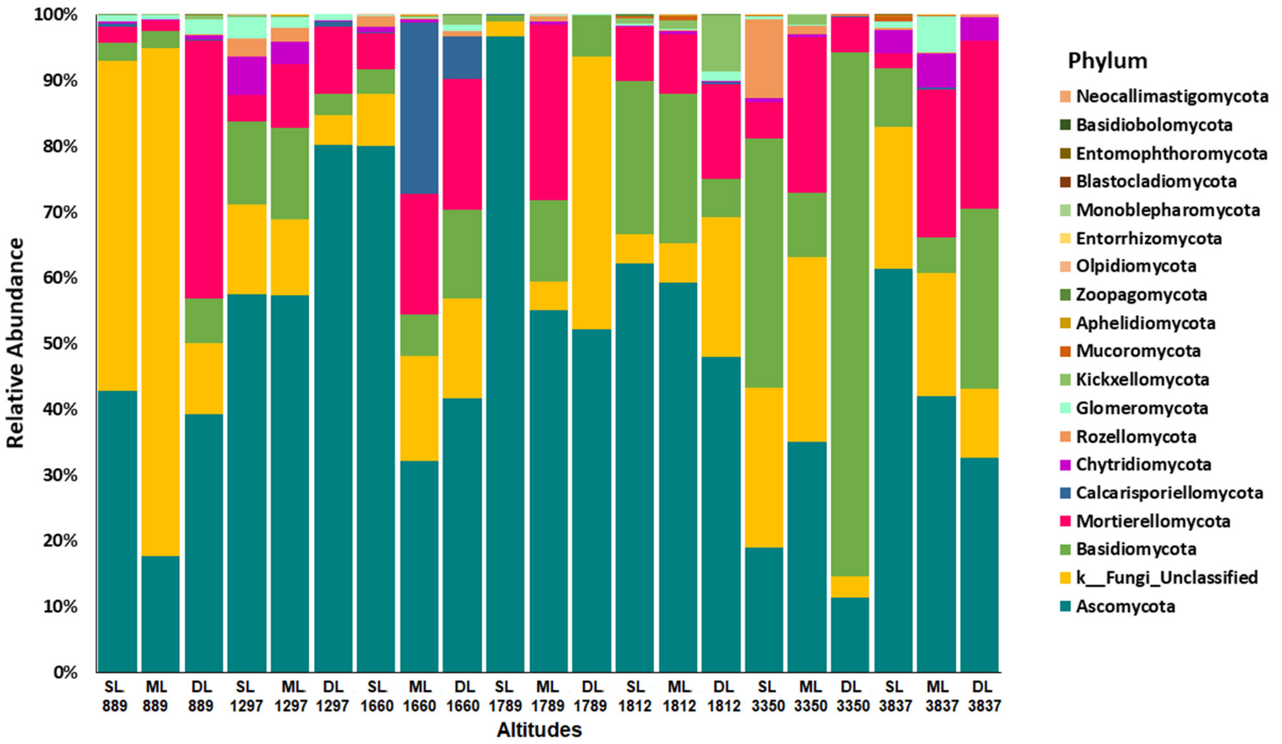

Figure S2. The relative abundance of fungal phyla along with elevation gradients and soil layers. SL: Surface Layer, ML: Middle Layer, DL: Deeper Layer.

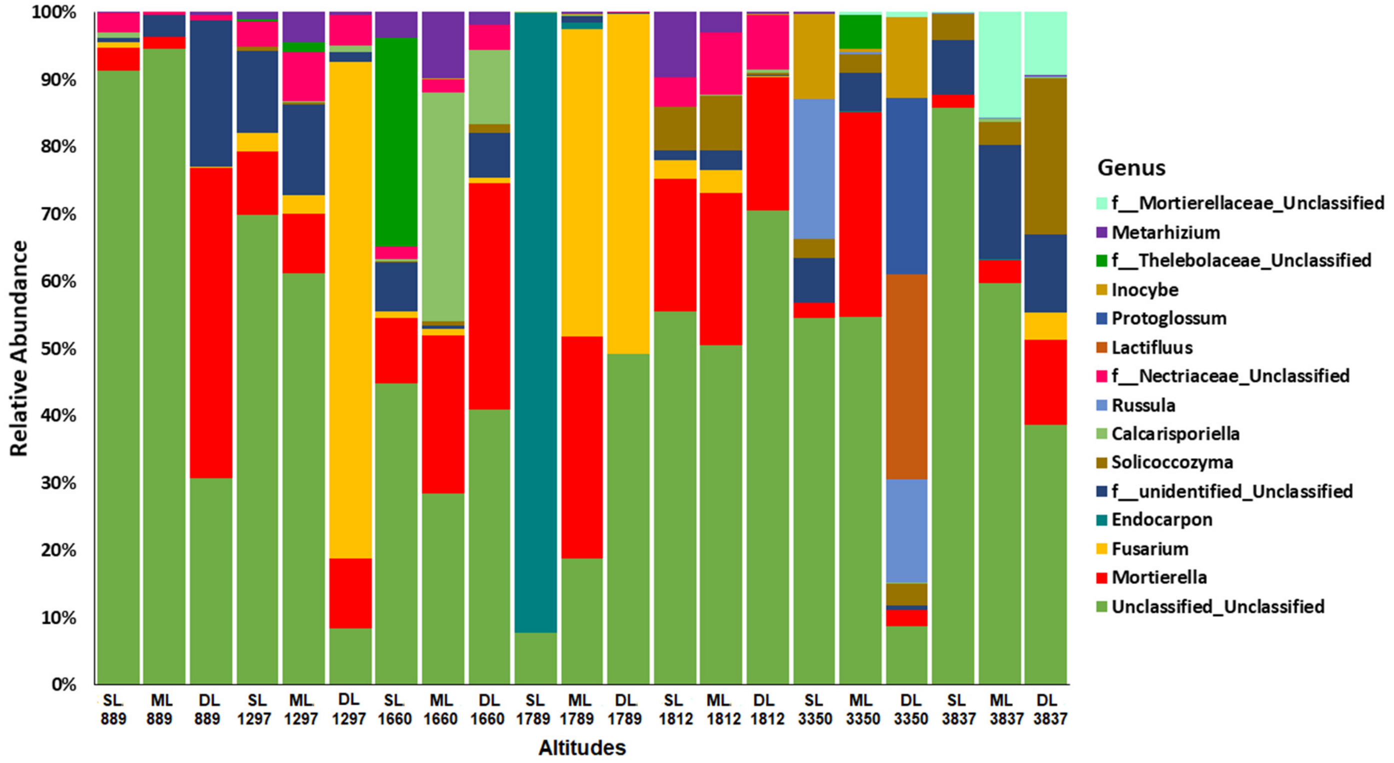

Figure S3. The relative abundance of top 15 fungal genera along with elevation gradients and soil layers.

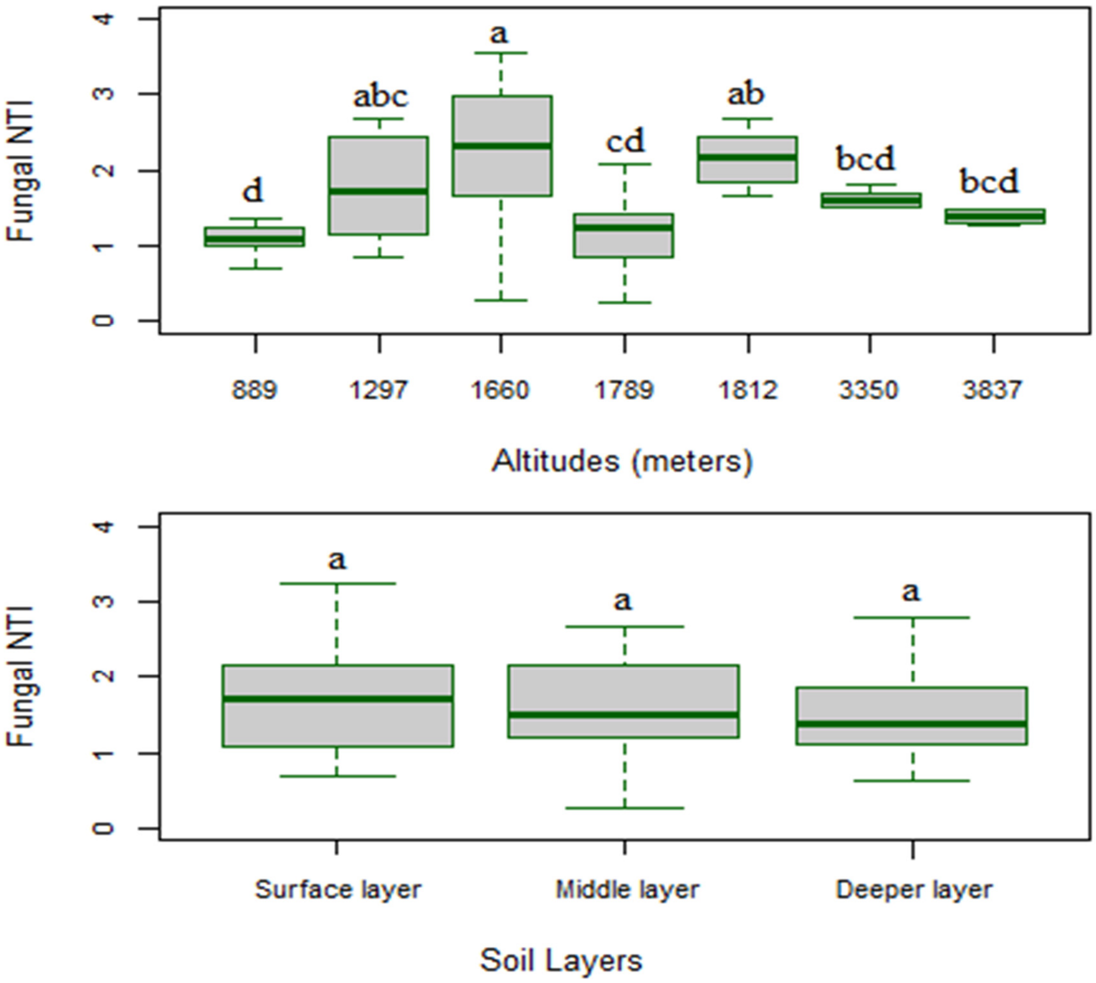

Figure S4. (**A**) Boxplot of Nearest Taxon Index (NTI) values of fungal communities along altitudinal gradients and (**B**) soil layers. The superscripts of different small letter denoted significant difference among all altitudes and soil layers (Duncan's test, $P < 0.05$).

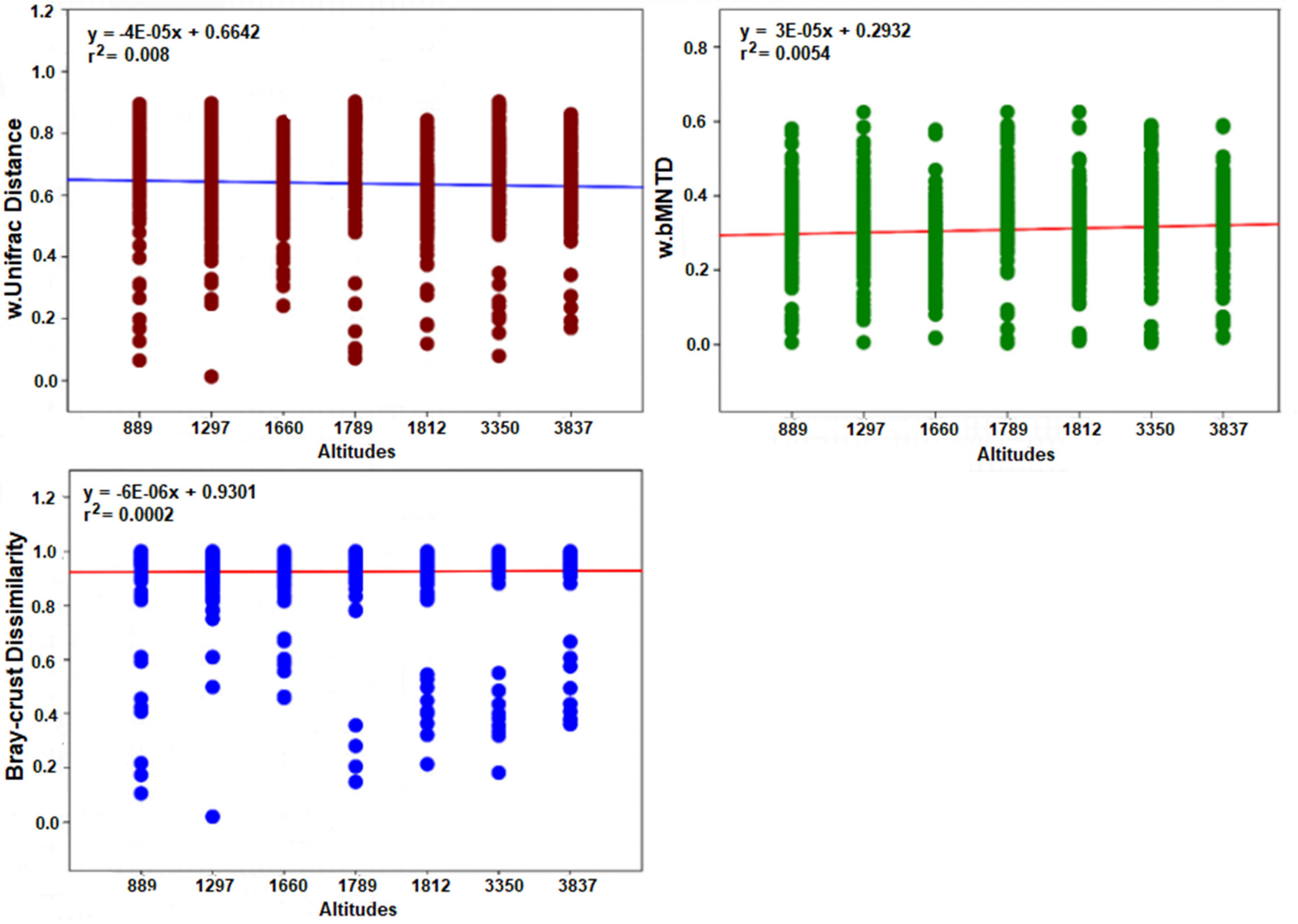

Figure S5. Linear regression analysis of fungal taxonomic and phylogenetic beta diversity indices among altitudinal gradients.

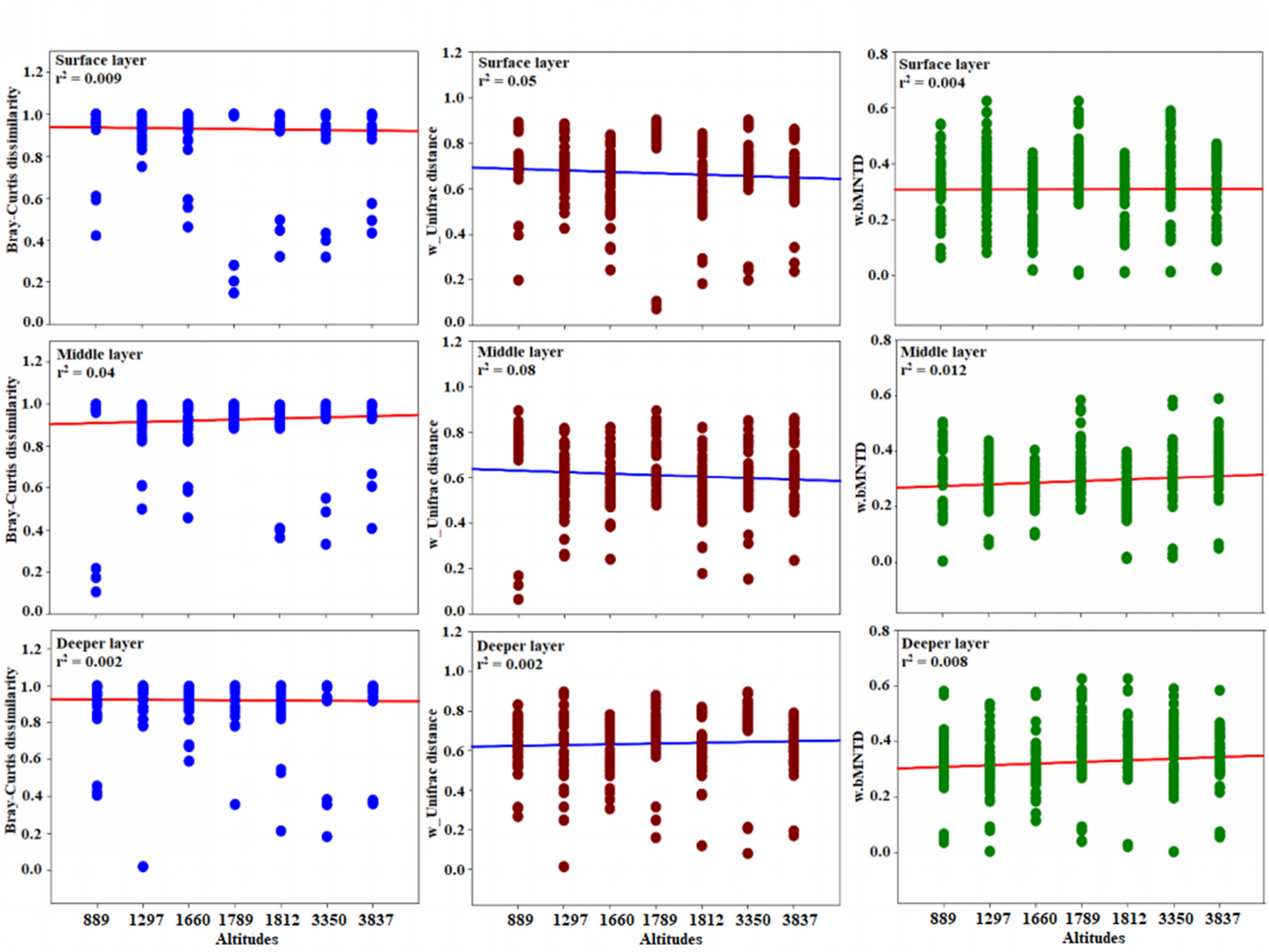

Figure S6. Linear regression analysis of fungal taxonomic and phylogenetic beta diversity indices among soil layers and altitudinal gradients.

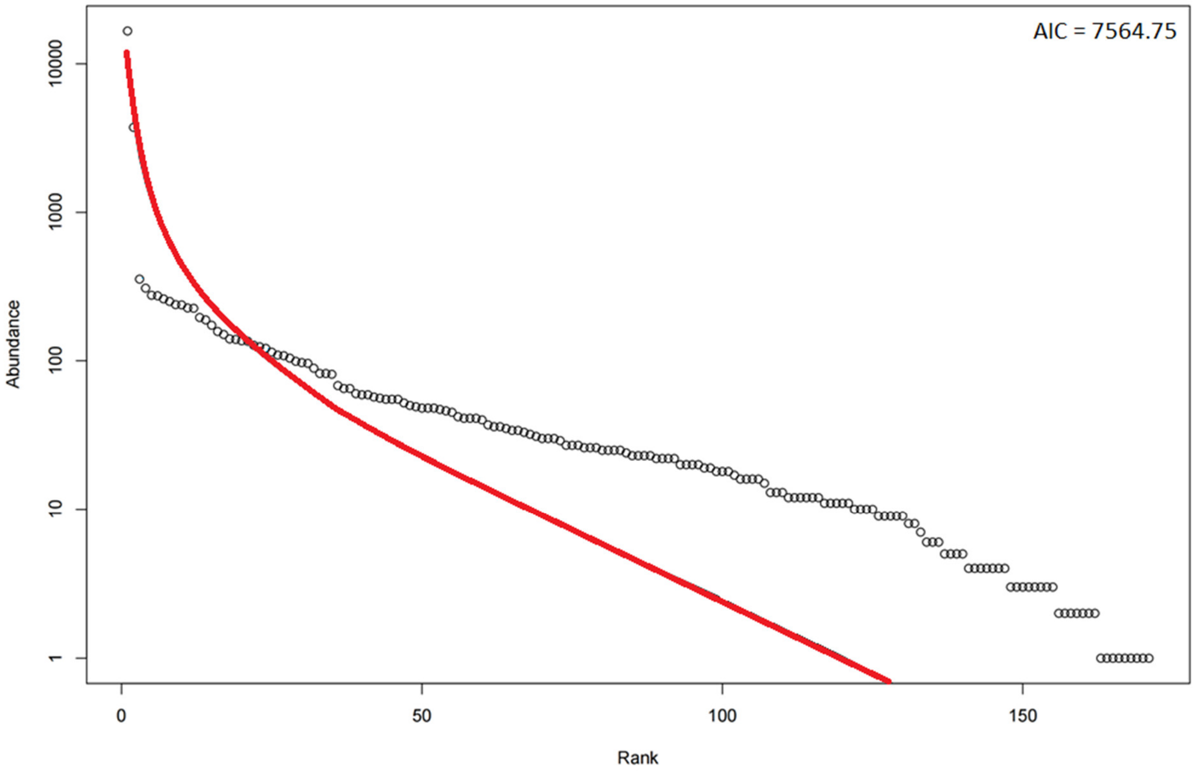

Figure S7. The rank abundance distribution lognormal model based on Akaike Information Criterion (AIC). The letters above boxes indicate significant differences between samples (Duncan's test, $p < 0.05$).
